# Incretins (GLP-1 Receptor Agonists) and Polycystic Ovaries

**DOI:** 10.5935/1518-0557.20250162

**Published:** 2025

**Authors:** Mauri José Piazza

**Affiliations:** 1 Tocogynecology Department, Federal University of Paraná - UFPR - Curitiba (PR), Brazil

**Keywords:** polycystic ovaries, metabolic syndrome, GLP-1 agonists, insulin resistance, semaglutide, liraglutide

## Abstract

Polycystic ovary syndrome (PCOS) is a widespread condition affecting women of reproductive age. A new class of medications called incretins (GLP1RAs) provides new opportunities for reducing obesity, hyperandrogenism, and insulin resistance in these patients. Promising results have been achieved, including metabolic improvements and benefits in women with PCOS using different GLP1RAs.

## POLYCYSTIC OVARIES

Polycystic ovary syndrome (PCOS) is the most common endocrinopathy affecting women of reproductive age. Stein & Leventhal (1935) described a complex clinical condition characterized by oligo-amenorrhea, hirsutism, and obesity associated with enlarged ovaries. Prevalence varies depending on different criteria and ranges from 6% to 15% or 20% (Ehrmann, 2005; Mayer *et al.*, 2015). The diagnostic criteria established in the Rotterdam Meeting in 2003 were later revised (The Rotterdam ESHRE/ASRM-sponsored PCOS consensus workshop group, 2004a, 2004b). PCOS involves menstrual disorders, oligo-amenorrhea, hyperandrogenism, insulin resistance, and obesity, sometimes including metabolic syndrome and infertility.

## MENSTRUAL DISORDERS

The most common menstrual abnormality is oligo-amenorrhea caused by chronic anovulation. Patients experience asynchronous production of gonadotropins, androgens, and estrogen. Symptoms and signs vary depending on the hormonal levels in the gonads, including signs of hirsutism. Other menstrual irregularities, such as endometrial hyperplasia and dysfunctional uterine bleeding, can also occur.

## HYPERANDROGENISM

In patients with chronic anovulation, the average daily production of estrogen and androgen is increased and depends on LH stimulation. Elevated levels of testosterone, androstenedione, dehydroepiandrosterone (DHEA), and dehydroepiandrosterone sulfate (DHEAS) (Baracat & Soares Jr, 2007; Teede *et al.*, 2023) were observed. Androstenedione and testosterone are produced in the ovaries, while dehydroepiandrosterone sulfate is made in the adrenal gland.

Insulin resistance is a hallmark of PCOS, affecting 50%-80% of patients (Diamanti-Kandarakis & Dunaif, 2012). The exact mechanism behind insulin resistance is controversial but seems to involve excessive serine phosphorylation and reduced tyrosine phosphorylation. The pancreas may become overstimulated, leading to hyperinsulinism (Dong *et al.*, 2019). The incidence of Type 2 diabetes has increased approximately threefold in patients with PCOS (Macut *et al.*, 2017).

Obesity is a chronic and multifactorial disorder that can result in various complications such as cardiovascular diseases, diabetes mellitus type 2, sleep apnea, and malignancies (Frühbeck *et al.*, 2019). It can cause significant metabolic abnormalities affecting individuals with PCOS. Prevalence in some groups may reach 50% to 70% (Ehrmann, 2005; Mayer *et al.*, 2015). Adolescents with a high body mass index (BMI) are at increased risk of developing PCOS (Frühbeck *et al.*, 2019). Women with PCOS often show elevated BMI and waist circumference, along with dyslipidemia and insulin resistance.

Obesity is often linked to three hormonal changes:

1. Increased peripheral aromatization of androgens into estrogens.
2. Decreased levels of sex hormone-binding globulin (SHBG) and increased levels of androgens and estrogens.
3. Increased insulin levels and stimulation, and increased androgens by ovarian stroma.

Cardiovascular Dysfunction - The link between obesity and insulin resistance may contribute to the development of plurimetabolic syndrome (Otaghi *et al.*, 2019). PCOS patients can experience cardiovascular disorders such as atherosclerosis, coronary diseases, and hypertension (Meyer *et al.*, 2012).

## AN OVERVIEW OF INCRETINS AND GLP1-RECEPTOR AGONISTS

Incretins are gut-endocrine hormones produced in the intestinal L cells of the ileum and proximal colon, released into the bloodstream shortly after a meal. Their function is regulated by insulin release and involves stimulating beta cell proliferation in the pancreas. Many incretins exist, but the most important are GLP (glucose-dependent polypeptide) and GLP-1 receptor agonists (GLP-1RAs). Their plasma levels increase after consuming carbohydrates, fats, and proteins. Endogenous GLP-1 has a very short half-life of 1-2 minutes and is broken down by the enzyme dipeptidyl peptidase 4 (DPP-4); GLP-1 itself lasts about 5 minutes. DPP-4 inhibitors are a class of oral anti-diabetic drugs that help regulate and control blood glucose levels.

A group of new medications based on GLP1RAs was recently developed according to indication, route, and frequency of administration. They were previously used only as injectables for managing type 2 diabetes mellitus. Today, other drugs with different indications are available in oral forms (Kalra *et al.*, 2021). They may be used for weight reduction, lowering cardiovascular risk, non-alcoholic steatohepatitis, sleep apnea, and polycystic ovary syndrome.

GLP1 and analogs are designed to stimulate insulin secretion, delay gastric emptying, inhibit glucagon production by pancreatic alpha cells, and increase satiety to regulate appetite. Other treatment indications for GLP1 or receptor analogs include a BMI over 30 kg/m2 or 27 kg/m2 with at least one weight-related comorbidity such as hypertension, dyslipidemia, type 2 diabetes, or sleep apnea (Cena *et al.*, 2020).

The side effects of GLP-1 are mainly gastrointestinal, including nausea (16-44%), vomiting and diarrhea (9-30%), constipation (3-24%), and abdominal pain (6-20%), with an occurrence of 1 in 10; however, further assessment is required. GLP-1 contraindications include pregnancy, breastfeeding, and a known genetic risk for medullary thyroid cancers or related multiple endocrine neoplasia type 1. Caution is recommended for individuals with a personal history of pancreatitis.

Short-acting drugs taken daily, such as Exenatide (Byetta), Liraglutide (Victoza), and Dulaglutide (Trulicity), are approved for treating Type 2 Diabetes. Semaglutide and Tirzepatide are administered subcutaneously. The starting dose of Semaglutide is 0.25 mg for 4 weeks, then increased to 0.5 mg. If needed, it can be further increased to 1-1.7 mg or 2.4 mg per week. Tirzepatide begins at 2.5 mg to 5 mg for 4 weeks. If weight loss occurs, the dose does not need to be increased.

## GLP1 RECEPTOR ANALOGS

Kalra *et al.* (2021) proposed a modern classification that included drug indication, route, and frequency of administration. New drugs were developed, others were maintained, while some were discontinued (Tables 1 and 2).

**Table 1:** Contemporary Classification of GLP1RAs.

MONOFORMULATIONS		
**ROUTE**	**INDICATION**	
	**Type 2 Diabetes mellitus**	**Obesity**
Oral	Semaglutide 3,7,14mg Semaglutide 25-50mg Danuglipron	Semaglutide 50mg Danuglipron (in develop)
Subcutaneous daily	Lixisenatide, Liraglutide 0.6-1.8mg	Liraglutide 3.0mg
Subcutaneous weekly	Exenatide LAR, Dulaglutide, Semaglutide 0.25-1.0mg	Semaglutide 2.4mg
Implantable	ITCA 650 I (discontinued)	
**CO-FORMULATIONS**		
**ROUTE**	**CONTENT**	
	**GLP1RA + insulin**	**GLP1RA + other agonist**
Subcutaneous daily	IDegLira; LixiLan	
Subcutaneous weekly	IcoSemaa	Sema-Amylin HM15211
		Tirzepatide 5, 10, 15mg

From: Kalra S, Bhattacharya S, Kapoor N. Contemporary Classification of Glucagon-Like Peptide 1 Receptor Agonists. Diabetes Ther. 2021;12:2133-47.

**Table 2:** Different Products and Comparison between GLP1RAs.

Drug	Brand Name	Via Adm	Dose	Half- Life T	Half- Life T	Approved
Dulaglutide	Trulicity	Subcutaneous	0.75, 1.5	5 days	once/week	2014
Semaglutide	Ozempic Rybelsus	Subcutaneous Oral	0.25, 1mg 3,7,14 mg	1 week 1 week	once/week once daily	2017
Liraglutide	Victoza Saxenda	Subcutaneous Subcutaneous	0.6,1.8mg 6mg	13h 3 days	once daily twice/week	2010
Exenatide ER	Bydureon BC Byetta	Subcutaneous Subcutaneous	2, 5,10µg 5,10µg	1 week 3h	once/week once daily	2005
Tirzepatide	Mounjaro Zepbound	Subcutaneous Subcutaneous	5mg 5,10,15mg	5 days 5 days	once/week once/week	2022 2023

From: Kalra S, Bhattacharya S, Kapoor N. Contemporary Classification of Glucagon-Like Peptide 1 Receptor Agonists. Diabetes Ther. 2021;12:2133-47.

Dulaglutide is an analog with a half-life of 4 days. The medication, branded Trulicity, is used as a once-weekly dose (Fahrbach *et al.*, 2016). The administration route is subcutaneous, at 0.7/1.5 mg per week. Semaglutide comes in tablets of 3, 7, or 14 mg, administered orally. The medication is branded Ozempic. The initial dose is 3 mg, with 7 or 14 mg used for maintenance. It has shown significantly better efficacy for type 2 diabetes (Knudsen & Lau, 2019). Semaglutide QW injections are administered once a week. Wegovy, 2.4 mg, is the brand name for a receptor agonist anti-obesity medication (Knudsen & Lau, 2019). Liraglutide has two brand names: Victoza for T2DM, at 0.6-1.8 mg with a half-life of 13 hours; and Saxenda, 6 mg for obesity, administered once daily, both given subcutaneously (Rigato & Fadini, 2014).

Exenatide BID has two brand names: Byetta 5-10 μg, with a half-life of 3 hours, administered once a day; and Bydureon BC 2,5-10 μg, administered once weekly as a long-acting formulation. Exendin was a peptide found in the saliva of the Gila monster, but Exenatide is a synthetic formulation (Szayna *et al.*, 2000). The brand names of Lixisenatide are Adlyxin (USA) and Lyxumia (Europe), and they are administered subcutaneously once daily at 10-20mg, with a half-life of 2-3 hours. It is derived from a foreign peptide, and an abnormal antibody response can occur (Barnett, 2011).

Tirzepatide has two brand names: Mounjaro 5mg, which has a half-life of 5 days and is used to treat type 2 diabetes, and Zepbound 5-10-15 mg for anti-obesity purposes. Both are administered subcutaneously. The former medication may increase the risk of developing thyroid tumors (Eli Lilly and Company, 2025). Albiglutide is a long-acting GLP-1 receptor agonist with two brand names: Eperzan and Tanzeum, both administered subcutaneously. The maximum concentration is reached after 3-5 days post-dosing, and its half-life is 4-7 days. Both drugs tend to have fewer gastrointestinal side effects (Rendell, 2016). was developed as an oral small molecule GLP-1 receptor agonist but was discontinued due to high rates of adverse effects (Griffith *et al.*, 2022).

## OTHER COFORMULATIONS

Many other efforts have been made with GLP1RA and insulin combinations, although the outcomes need evaluation.

Associations:

1. iGlar U-100/Lixisenatide 33 or 50µg/ml (iGlarLixi)
2. Insulins Degludec 100U/ml and Liraglutide 3.6mg/ml (IDegLira)
3. Icosema: Insulin Icodec 350 U/Semaglutide.

DPP-4 (DIPEPTIDYL PEPTIDASE 4) is an enzyme that regulates and inhibits the production of GLP-1 in the ileum and colon.

Oral tablets:

Sitagliptin: Brand name - Januvia, 100 mg/day, was the first DPP-4 inhibitor.

Alogliptin: Brand names - Nesina and Vipidia; dose range - 6, 12.5, and 25 mg/day; DPP-4 inhibitor.

## EFFECTS OF GLP-1 ON POLYCYSTIC OVARY SYNDROME

PCOS is a prevalent disorder that causes hormonal abnormalities and obesity, along with hyperinsulinemia and insulin resistance. At the same time, higher insulin levels lead to increased free androgen levels by reducing the production of Sex Hormone-Binding Globulin (SHBG) (Xing *et al.*, 2022) and contribute to weight gain. GLP-1 Receptor Agonists such as Liraglutide, Exenatide, and Semaglutide have emerged as a new class of drugs with specific advantages for treating metabolic disorders. They are a novel therapeutic option for PCOS.

Insulin resistance in patients with PCOS occurs independently of body weight, and inherited post-receptor defects can be identified, affecting insulin target tissues such as the liver, skeletal muscle, and adipose tissue. Jensterle *et al.* (2015a) reported a greater BMI reduction with daily Liraglutide administration of 1.2 mg compared to 1000 mg of Metformin twice daily (reduction of 1.1-1.26 kg/m^2^ for Liraglutide *versus* 0.1-0.67 kg/m^2^ for Metformin). Liraglutide treatment significantly decreased visceral adipose tissue area. In another study with 30 obese women with PCOS, Jensterle *et al.* (2017a) evaluated a smaller dose of Liraglutide: 1.2 mg combined with Metformin *versus* a high dose of Liraglutide: 3 mg alone. Treatment with the higher dose of Liraglutide alone proved superior to the lower doses combined with Metformin.

Frøssing *et al.* (2018) enrolled 72 overweight women with PCOS who were treated with Liraglutide 1.8 mg/day or a placebo for 26 weeks. The trial showed that treatment with Liraglutide significantly reduced body weight by over 5%, visceral adipose tissue by 18%, and free testosterone levels by 19%. Other studies involving Liraglutide were conducted by Helvaci & Yildiz (2023), Papaetis & Kyriacou (2022), Baranowska-Bik (2022), Tilinca *et al.* (2021), Astrup *et al.* (2012), Wadden *et al.* (2013), Pi-Sunyer *et al.* (2015), Nylander *et al.* (2017a, 2017b), Elkind-Hirsch *et al.* (2022). Liraglutide is used as a first-line anti-obesity medication and was approved for treating type 2 diabetes at a maximum dose of 1.8 mg/day subcutaneously. However, in the SCALE program (Satiety and Clinical Adiposity-Liraglutide Evidence), the dosage was increased to 3 mg/day (Wadden *et al.*, 2013).

Losing body weight improves control of hyperandrogenism, reproductive function, and metabolic issues in women with PCOS. In a 12-week randomized study conducted by Jensterle *et al.* (2015a), 45 women with PCOS and obesity were treated with Metformin 1000 mg or Liraglutide 1.2 mg daily. Liraglutide showed a significant reduction in BMI compared to Metformin, including a notable decrease in visceral adipose tissue. A meta-analysis of seven randomized studies (Elkind-Hirsch *et al.*, 2008; Jensterle Seve *et al.*, 2014; Rasmussen & Lindenberg, 2014; Jensterle *et al.*, 2015a; 2015b; 2015c; Kahal *et al.*, 2015) confirmed Liraglutide’s effectiveness (Niafar *et al.*, 2016). Other effects of GLP1RAs on reproductive outcomes and PCOS were described in clinical studies.

## OVARIAN CHARACTERISTICS AND OVULATION RATE

Ultrasound images of obese PCOS patients treated with Liraglutide over six months showed a significant reduction in ovarian volume compared to placebo (Nylander *et al.*, 2017a). No improvement was seen in the Androgen Ferriman-Gallwey scale for hirsutism characteristics. Bednarz *et al.* (2022) found that GLP1 may have anti-inflammatory and antifibrotic effects on the gonads and endometrium while reversing polycystic ovary morphology and lowering serum androgen levels. Elkind-Hirsch *et al.* (2017) studied 60 overweight and obese patients treated with Exenatide 10 µg and Exenatide 10 µg plus Metformin 1000 mg and observed an increase in ovulation rates in the latter group.

## MENSTRUAL REGULARITY

A study involving 60 overweight and obese women with PCOS was conducted over 24 weeks. The participants were divided into two groups: one received Exenatide 10µg twice daily, and the other received Exenatide 10µg combined with Metformin 1000mg twice daily. The second group showed improved menstrual regularity (Elkind-Hirsch *et al.,* 2017). Another study with 40 obese women with PCOS divided subjects into two groups: one given Liraglutide 1.2mg once daily and the other using Liraglutide 1.2mg combined with Metformin 1000mg (Jensterle Seve *et al.,* 2014). Menstrual regularity did not change. In another study (Jensterle *et al.*, 2015a; 2015b), 32 and 41 obese women with PCOS were treated for 12 weeks. The first group received Liraglutide 1.2mg once daily, while the second group received Liraglutide plus Metformin 1000mg twice daily. Menstrual effects remained unchanged.

Kahal *et al.* (2015) evaluated 19 obese women with PCOS *versus* 17 obese controls treated with Liraglutide 1.8mg QD for 24 weeks. In both groups, menstrual results remained unchanged. Nylander *et al.* (2017a) observed 72 patients with PCOS treated with Liraglutide 1.8mg QD *versus* placebo for 24 weeks. Menstrual cycle regularity was good. Elkind-Hirsch *et al.* (2017) assessed 34 women with PCOS and prediabetes treated with Saxagliptin 5mg *versus* Metformin 2000mg for 16 weeks. The authors noted improved menstrual cycle regularity. Jensterle *et al.* (2017b) observed 30 women with PCOS and insulin resistance, despite treatment with Metformin 2000mg. They were treated with Alogliptin 25mg + Pioglitazone 30mg + Metformin 2000mg *versus* Alogliptin 25mg + Metformin 2000mg for 12 weeks, showing better menstrual cycle regularity.

## PREGNANCY RATE

The pregnancy rate per embryo transfer was higher among subjects given Liraglutide than those treated with Metformin alone. Liu *et al.* (2017) assessed 176 overweight and obese women with PCOS over 12 weeks, treated with Exenatide 10 µg BID (Group 1) or Metformin 1000 mg BID (Group 2). In both groups, the natural pregnancy rates were similar. Salamun *et al.* (2018) analyzed 28 obese women with PCOS over 12 weeks. Group 1 received Liraglutide 1.2 mg QD plus Metformin 1000 mg BID, while Group 2 received only Metformin 1000 mg BID. Pregnancy rates exceeded those seen in the In Vitro Fertilization group. Yang & Wang (2016) described the case of an infertile woman with PCOS and obesity treated with Exenatide 10 µg BID for 8 weeks who became pregnant after treatment.

## LIVE BIR

There are concerns about the safety of GLP-1 during the first trimester, throughout pregnancy, and in postpartum lactation. Novo Nordisk’s safety assessment included 271 cases of Liraglutide use during pregnancy. Of these, 45.9% resulted in live births without congenital anomalies, 1.8% in live births with congenital anomalies, 34.2% in fetal losses, and 18% in pregnancy terminations (Center for Drug Evaluation and Research, 2017). All patients treated with Semaglutide had healthy children, with no pregnancy losses or fetuses with congenital anomalies (Muller *et al.,* 2023). Greco (2015) described the case of a woman with type 2 diabetes mellitus and PCOS treated with Liraglutide 1.8 mg QD during the first trimester of pregnancy who had a healthy newborn.

If maintained during pregnancy, GLP-1RAs can be used after birth. There is no conclusive evidence of GLP-1 in breastmilk. Manufacturers of Semaglutide recommend avoiding the medication for 8 weeks before pregnancy, and Tirzepatide for 4 weeks prior to pregnancy. Preclinical animal studies indicate higher risks of early pregnancy loss, lower fetal weight, and visceral malformations (FDA, 2025). Currently, six FDA-approved medications for weight loss are available: two GLP-1RAs (Liraglutide and Semaglutide); one dual GLP-1 and gastric inhibitory peptide (GIP/GLP-1) receptor agonist (Tirzepatide). Other options, including Contrave (naltrexone/bupropion), Qsymia (phentermine/Topamax), and Xenical (orlistat), have also been approved (Duah & Seifer, 2025).

## CONCLUSION

Our goal with this paper was to review and discuss publications by various authors on the use of GLP and GLP-1 to treat patients with Polycystic Ovary Syndrome, focusing on different medication doses, durations, and indications. The initial results with GLP-1RAs were promising in overweight and obese patients with PCOS. However, further studies are needed to reach more definitive conclusions.
